# A Manual of Examinations, upon Anatomy and Physiology, Surgery, Practice of Medicine, Chemistry, Materia Medica, Obstetrics, &c.

**Published:** 1844-10

**Authors:** 


Art. XI.—*A Manual of Examinations, upon Anatomy and Physiology, Surgery, Practice of Medicine, Chemistry, Materia Medica, Obstetrics, &c.* Designed for the use of Students of Medicine throughout the United States. By J. L. Ludlow, M.D., Philadelphia: Ed. Barrington & Geo. D. Haswell. 1844. 12mo. pp. 615.

This volume ought to be classed among *labor-saving* machines. It is admirably calculated to save physicians the trouble of thinking. It contains all the questions likely to occur in medical examinations, with the answers thereto, and consequently does not require in the examiner any knowledge of medical subjects. We should think a book of this sort might prompt many gentlemen to examine their students, who now never put a medical question to them during the whole period of their pupilage. And we are quite sure that it will be found a very convenient aid to others, who feel the evening examinations to be a great bore, after the exhausting labors of the day. Between all the classes whose comfort the *Student's Manual* is designed to promote, we should think it would obtain not a little currency. As a sort of 'short cut' to knowledge, it is sure to command the regard of students, and these will find it, we have no doubt, a convenient and useful manual. The author has brought within a small compass a large mass of information on all the subjects of medicine.

